# Inpatient rehabilitation service utilization and outcomes under US ACA Medicaid expansion

**DOI:** 10.1186/s12913-021-06256-z

**Published:** 2021-03-20

**Authors:** Ying ( Jessica) Cao, Jing Nie, Katia Noyes

**Affiliations:** 1 Department of Population Health Sciences, University of Wisconsin – Madison, 760B WARF Office Building, 610 Walnut St, Madison, WI 53726 USA; 2 Department of Epidemiology and Environmental Health, Division of Health Services Policy and Practice, The State University of New York – Buffalo, Buffalo, NY USA

**Keywords:** Medicaid, Post-acute care, Health outcomes and functional status, Care access and utilization

## Abstract

**Background:**

To investigate the impact of the US Medicaid expansion on care utilization and health outcomes of patients treated in the inpatient rehabilitation facilities (IRF).

**Methods:**

A retrospective observational study with a difference-in-difference design. The data was obtained from Inpatient Rehabilitation Facility – Patient Assessment Instrument (IRF-PAI). Sample included all Medicaid beneficiaries (aged 18–64 years) who received initial inpatient rehabilitation for stroke, hip fracture (acute conditions), or joint replacement (elective condition) (*N* = 14,917) before (2013) and after (2016) the expansion. The study estimated the differences in length of stay, functional improvement, and possibility of returning to community before and after ACA Medicaid expansion in the expansion regions relative to the non-expansion regions. The analysis was fully adjusted for patient demographics, health conditions, facility characteristics and time trends.

**Results:**

Compared with non-expansion states, service volume in the expansion regions increased more for the two acute conditions (49 and 27% vs. 1% and − 4%) and decreased less for the selective condition (− 12% vs. -34%) after ACA Medicaid expansion. Medicaid expansion was associated with significant decreases in patient functional improvements (− 1.63 points for stroke, − 3.61 points for fracture and − 2.73 points for joint; *P* < 0.05). Length of stay and the possibility of returning to community after discharge were not significantly different.

**Conclusions:**

Medicaid expansion was associated with increases in the utilization of inpatient rehabilitation services and decreases in the patient functional improvements. Cautions should be taken with the decreases in functional improvements among some subpopulation in the short-term; longer follow up periods are needed to account for gradual changes in patient needs.

## Background

Medicaid in the United States is a federal and state program that helps with medical costs for some people with limited income and resources [1]. The Patient Protection and Affordable Care Act (PPACA or ACA) significantly expanded both eligibility for and federal funding of Medicaid [2]. Under the PPACA, many states expanded Medicaid coverage to individuals with incomes up to 138% of Federal Poverty Level (FPL) effective on January 1, 2014. Some other states chose to expand at later dates while the remaining ones chose not to expand. As of November 2018, 37 states have opted to expand Medicaid eligibility, 30 of which made the coverage effective before January 1, 2016 (2 states started during 2016, 1 in 2019, and 4 to be determined) [1]. Research estimating the effects of Medicaid expansion on the original goals of ACA found that the expansion was generally associated with increases in coverage, service utilization, quality of care and Medicaid spending in preventive, primary, acute and emergency care settings [3–8]. Mazurenko et al. [9] and Anotnisse et al. [10–12] provided systematic reviews on these topics.

Post-acute care (PAC) provides continued recovery from illness or health shocks to restore physical and cognitive functionality. PAC plays essential roles to prevent functional deterioration and maintain life independence and quality [13]. It is estimated that in the United States approximately 42% of all hospitalized Medicaid and Medicare beneficiaries received PAC services after discharge from an acute care facility in 2015 [1, 14]. Within the past decade, due to the higher growth in costs comparing with the other spending categories, PAC admission and reimbursement requirements became more stringent, forcing more patients to choose less costly home care and/or skilled nurse facilities instead of costlier inpatient rehabilitation facilities (IRF) [2, 13]. There are concerns about access and quality of care delivery in the whole PAC sector in general and among the IRF in particular [2, 15].

Very few studies have focused on inpatient rehabilitation services under the ACA Medicaid expansion reforms or the general background of the steep decline in inpatient admissions among IRF [2, 13] in the recent years. Those that are focused on IRF with Medicaid expansions were limited to trauma and child patient populations [16, 17]; thus far, no study has focused on non-elderly adults with major rehabilitation admission conditions, e.g., stroke, hip fracture and joint replacement, etc.

The objective of this study is to investigate the effect of Medicaid expansion under ACA on PAC utilization, delivery and patient health outcomes among non-elderly adults in IRF. First, the study will estimate the PAC utilization and outcome changes before and after the policy reform – Medicaid expansion among all the Medicaid beneficiaries. Second, the study will investigate whether the policy effects vary by the patient admission conditions and/or the existence/severity of comorbidities. Third, in order to isolate the time varying unobservable effects that are not due to the Medicaid expansion, this study will adopt a difference-in-difference (DinD) design and use the non-expansion states before and after the ACA reform (i.e., the expansion) as a comparison group.

## Methods

### Study design

This study used a difference-in-difference (DinD) design to estimate the effects of Medicaid expansion on post-acute inpatient rehabilitation service utilization and health outcomes by comparing the expansion states and non-expansion states before and after the 2014/2015 Medicaid expansion policy implementation. The year 2013 was used as the pre-expansion baseline year and 2016 was used as the post-expansion target year. Expansion states were defined as those states which implemented Medicaid expansion before January 1, 2016, while the states that did not expand their Medicaid programs until after January 1, 2016 were defined as non-expansion states. By incorporating the non-expansion states in the analysis for comparison, the difference-in-difference method controlled for the effects of time variant unobservable using the changes of outcome variables in the non-expansion states before and after the policy reform (i.e., Medicaid expansion) [4, 5, 8].

### Data sources

This study, approved by the Institutional Review Board of the State University of New York at Buffalo (Reference No.: STUDY00001563), used data from the Uniform Data System for Medical Rehabilitation (UDSMR). UDSMR maintains the world’s largest non-government data repository for inpatient medical rehabilitation in the United States and covers about 80% of the IRF in the industry [18, 19]. Inclusion in UDSMR is primarily based on historical business administrative decisions unrelated to geographic area or facility type [18]. UDSMR uses the Inpatient Rehabilitation Facility - Patient Assessment Instrument (IRF-PAI) to document patient records. It includes sociodemographic variables, diagnoses (International Classification of Diseases Ninth Revision [ICD-9] codes), IRF characteristics, discharge disposition, and cost factors such as length of stay (LOS), source of payment, and facility charges [18, 19]. In addition, IRF-PAI also documents patient functional measures of basic daily living using the Functional Independence Measure (FIM instrument). The Center for Medicare & Medicaid Services (CMS) adopted UDSMR’s FIM instrument for the IRF-PPS (IRF Prospective Payment System) [20].

### Study sample

The study sample included all non-elderly adult (between the ages of 18 and 64) Medicaid beneficiaries who were admitted to IRF in 2013 (the reference year before Medicaid expansion) and 2016 (the comparison year after expansion) due to stroke, hip fracture and joint replacement based on UDSMR Impairment Group Codes (IGC) (01.1–9 for stroke; 08.11–12, 08.4 for hip fracture; and 08.51–52, 08.61–62, 08.71–72 for lower extremity joint replacement – hip and knee) (*N* = 17,508 patient records). These three groups were selected because they were the most common reasons for IRF admissions. Stroke and hip fracture were the two most common acute conditions and joint replacement was the most common elective condition. Selection of these 3 conditions also followed other rehabilitation studies targeting to the entire sector in general [21–23]. These three major health conditions jointly accounted for approximately 37% of all services delivered. Data from 2014 and 2015 were excluded because states and regions had different effective dates for Medicaid expansion throughout these 2 years, yielding a mixture of expansion and non-expansion cases in those years. Appendix Table 5 provided the implementation dates of Medicaid expansion by state, total beneficiaries before and after the expansion and the percentage share of patient records by the 10 CMS regions.

Further, a patient record was excluded if it was not an IRF admission for initial rehabilitation (e.g., transfers from other rehabilitation facilities, readmission, etc.), if the patient was living in a non-home setting before hospitalization, or if the patient died during the inpatient rehabilitation (*N* = 273). These patients generally had more complicated clinical situations that need additional adjustments or will bias the overall study sample [21–23]. Records with missing data on key outcome variables (e.g., FIM gain, discharge setting, etc.) (*N* = 8) and records with second payer listed as Medicare (*N* = 27) were also excluded, the latter of which were for patients covered by Medicare due to early-life disability and, therefore, deemed as incomparable to other patient records. The final sample included 14,917 patient records: 11,619 for stroke, 1270 for hip fracture and 2028 for joint replacement, which represents approximately 85% of the eligible sample.

### Outcome variables

#### Length of stay

Following from the team’s previously published study [19], length of stay (LOS) was calculated as the number of days spent in IRF excluding treatment or program interruptions (i.e., days when patients were absent from the facilities). LOS is proportional to treatment costs or per episode resource utilization [24–26].

#### Functional status

The patients’ functional status was measured by the FIM instrument within 3 days of admission and discharge. The FIM instrument consists of 18 items covering 6 domains measuring the motor and cognitive functions. The score ranges between 18 to 126 with higher scores indicating more independence [23, 26]. Detailed information regarding the FIM instrument can be found in the team’s previous study [19]. The FIM instrument has also been validated and used by many other studies in the field [24–27].

The FIM score at admission approximates the severity of a patient’s pre-treatment clinical condition. Changes in the mean value of FIM admission across years and subpopulations reflect the changes in the stringency of inpatient admission requirements (and hence, treatment accessibility) due to policy restrictions [2, 13].

#### Functional improvement

The functional improvement during an inpatient stay was calculated as the FIM score changes between admission and discharge. Larger functional improvement suggests better health outcomes or care quality. For any given functional improvement, longer LOS yields lower daily average functional improvment [26].

#### Discharge to community

Discharge setting was defined as a binary indicator – being able to return to community (i.e., home settings, =1) as opposed to transfer/discharge to another institutional setting (e.g., hospitals, long-term care, etc., =0) [23, 24, 26].

### Control variables

Control variables in the study included patient basic demographics (e.g., age, sex, race/ethnicity), socioeconomic status (e.g., marital status, geographic region, and insurance type - Medicaid fee-for-service or managed care), clinical factors (FIM at admission, CMS defined comorbidity tiers and case-mix group) and facility characteristics. Selection of these variables was based on the availability of the UDSMR dataset and other studies on rehabilitation outcomes using the same dataset [19, 24–27]. These four categories of control variables also resembled the general framework of determinants of population health covering factors in biology, socioeconomics, clinical conditions, health care policy and health care system/provider-level settings.

#### Clinical factors

In addition to the FIM score at admission, comorbidity tiers and case-mix group (CMG) were also used as clinical factors. The CMS tier system classifies the comorbidity levels – none, minor, moderate and major. CMS tiers reflect comorbidities that influence rehabilitation service use [18]. The CMG grouper is a classification system adopted by CMS to classify patients in IRFs for claims and reimbursement purposes. It used information from the IRF-PAI assessment to aggregate acute care inpatients that have similar clinical characteristics in order to determine appropriate resource use, costs and payments [18, 20]. Appendix Table 6 provided the detailed information on the classification of the alternative CMGs for the three conditions – stroke, hip fracture and joint replacement.

#### Facility characteristics

Facility characteristics such as number of certified beds and facility type (i.e., a stand-alone IRF vs. a unit within the same acute care facility) were included in the model to control the effects of facility-specific characteristics on the rehabilitation outcomes [24–27].

### Statistical models

The difference-in-difference models were specified as follows:

$$ {Y}_{ij}={\beta}_1{Expansion}_i+{\beta}_2{Post}_i+{\beta}_3{Expansion}_i\times {Post}_i+{X}_i\delta +{State}_j+{\epsilon}_i $$

where *Y*_*ij*_ denoted the outcome of interest (e.g., LOS, return to community, etc.) for patient i in state j. *Expansion* and *Post* were two dummy variables. *Expansion* = 1 if the patient received care in an expansion state, and 0 otherwise. *Post* = 1 if the patient received care in 2016 after the expansion, and 0 otherwise. The coefficient on the interaction term *β*_3_ was the difference-in-difference estimate which captured the pure treatment effect of Medicaid expansion (i.e., the 2nd difference) after controlling for the common time trend of pre- and post- differences using the non-expansion states (i.e., the 1st difference). *X*_*i*_ denoted the control variables, *State*_*j*_ was the state fixed effects, and *ϵ*_*i*_ was the residual.

Follow other studies using the same methods on Medicaid expansion [4, 5, 8, 28, 29], all estimates were obtained from linear (or linear probability) models, which allowed for direct interpretation of estimated coefficient as percentage point changes relative to the average (or mean probability) of the outcome variable of interest. In order to capture the differentiated effects of Medicaid expansion for the subpopulations with and without comorbidities, the study estimated the models separately for the two patient groups. The “seemingly unrelated estimation” (SUEST) method was used to compare the treatment effects (i.e. the difference-in-difference estimate) between the two groups [30]. All the analysis and results were adjusted for patient demographics, health conditions, facility characteristics, and regional fixed effects [21–23].

Further, sensitivity analysis was performed by excluding the patient admission records with treatment interruptions during the inpatient stays, discharges against medical advice, or atypical LOS (> 30 days or < 3 days); including patients with imputed values for missing variables (*N* = 8) and/or corrected payer classifications (*N* = 27); excluding the states with early expansion [7, 28, 29]; and using Medicaid regional enrollment numbers and percentage changes to re-weight the results. We also tested the requirements for linear regression, tried the alternative non-linear models such as discrete count and logistic regressions, etc. for the non-continuous outcomes, and compared between models with and without adjustments by the control variables. The results were robust to the model specifications, control variable and/or sample adjustments. We finally chose to present the results from the linear models adjusted by all the control variables for the purpose of convenient comparison with other studies on the same topic [4, 5, 8, 28, 29]. The analysis was performed by SAS 9.1 and significance of the results were reported at 90, 95 and 99% levels.

## Results

### Care accessibility and study population

Table 1 shows the study population for the two acute conditions (i.e., stroke and hip fracture) and one elective condition (i.e., joint replacement) by expansion and non-expansion states before and after the policy change. The total number of patients treated in the expansion states changed from 3621 in 2013 to 5410 in 2016 for stroke; from 340 to 432 for hip fracture; and from 709 to 626 for joint replacement. The service volume increased by 49.41, 27.35% and − 11.83% (decrease for joint replacement) respectively. During the same period, the number of patients treated in the non-expansion states changed from 1289 to 1303; from 254 to 244; and from 418 to 275 respectively, with the percentage changes calculated as 1.09, − 3.15% and − 34.21%. Compared with non-expansion regions during the same period, Medicaid expansion was associated with larger increases in service utilization for stroke and hip fracture – two non-deferrable acute medical conditions – and smaller decreases in care utilization for joint replacement – an elective procedure.

**Table 1:** Summary Statistics

	Non-Expansion States			Expansion States		
	2013	2016	*P* value*	2013	2016	*P* value*
Stroke						
N	1289	1303	~	3621	5406	~
Length of Stay (Days)	18.82 (14.04)	17.18 (13.00)	0.002	17.05 (11.62)	16.05 (10.78)	< 0.001
FIM Gain	29.79 (15.82)	32.12 (16.02)	< 0.001	28.77 (15.31)	29.70 (15.38)	0.005
LOS Efficiency	2.07 (1.75)	2.42 (2.18)	< 0.001	2.20 (1.81)	2.35 (1.81)	< 0.001
Return to Community (=1)	1012 (78.5%)	1062 (81.5%)	0.057	2667 (73.7%)	4036 (74.6%)	0.303
FIM Admission	52.24 (19.13)	53.87 (17.96)	0.026	55.62 (18.90)	57.10 (19.17)	< 0.001
Primary Payer (MCO = 1)	315 (24.4%)	661 (50.7%)	< 0.001	1646 (45.5%)	3289 (60.8%)	< 0.001
Case-Mixed Groups - 101 N (%)	38 (2.9%)	44 (3.4%)	0.636	144 (4.0%)	282 (5.2%)	0.002
CMG - 102	60 (4.7%)	71 (5.4%)		236 (6.5%)	402 (7.4%)	
CMG - 103	18 (1.4%)	26 (2.0%)		64 (1.8%)	104 (1.9%)	
CMG - 104	127 (9.9%)	137 (10.5%)		434 (12.0%)	744 (13.8%)	
CMG - 105	135 (10.5%)	127 (9.7%)		401 (11.1%)	558 (10.3%)	
CMG - 106	114 (8.8%)	139 (10.7%)		362 (10.0%)	516 (9.5%)	
CMG - 107	113 (8.8%)	108 (8.3%)		345 (9.5%)	461 (8.5%)	
CMG - 109	129 (10.0%)	122 (9.4%)		362 (10.0%)	484 (8.9%)	
CMG - 110	533 (41.3%)	511 (39.2%)		1216 (33.6%)	1742 (32.2%)	
CMG - 5001	22 (1.7%)	18 (1.4%)		57 (1.6%)	112 (2.1%)	
Comorbidity Tier						
- None	832 (64.5%)	696 (53.4%)	< 0.001	2486 (68.7%)	3138 (58.0%)	< 0.001
- Minor	366 (28.4%)	528 (40.5%)		946 (26.1%)	2012 (37.2%)	
- Moderate	23 (1.8%)	20 (1.5%)		45 (1.2%)	57 (1.1%)	
- Major	68 (5.3%)	59 (4.5%)		144 (4.0%)	203 (3.8%)	
Age (Years)	51.89 (9.71)	51.3 (9.91)	0.127	51.85 (9.44)	52.15 (9.4)	0.142
Gender (Female = 1)	667 (51.7%)	629 (48.3%)	0.077	1584 (43.7%)	2351 (43.5%)	0.81
Hispanic (=1)	66 (5.1%)	32 (2.5%)	< 0.001	580 (16.0%)	747 (13.8%)	0.004
Black (=1)	615 (47.7%)	508 (39.0%)	< 0.001	1087 (30.0%)	1617 (29.9%)	0.883
Marital Status						
- Married	362 (28.1%)	393 (30.2%)	0.673	1086 (30.0%)	1550 (28.7%)	< 0.001
- Never Married	528 (41.0%)	527 (40.4%)		1607 (44.4%)	2506 (46.3%)	
- Separated/Widowed	347 (26.9%)	332 (25.5%)		823 (22.7%)	1084 (20.0%)	
- Missing	52 (4.0%)	51 (3.9%)		105 (2.9%)	270 (5.0%)	
No. of Certified Beds	62.56 (40.76)	59.61 (42.46)	0.071	56.39 (47.64)	54.47 (40.56)	0.050
Facility type						
- Freestanding	540 (41.9%)	/	< 0.001	1169 (32.3%)	/	< 0.001
- In-Unit	749 (58.1%)	/		2452 (67.7%)	/	
- Missing	/	1303 (100.0%)		/	5410 (100.0%)	
Fracture						
N	254	244	~	340	432	~
Length of Stay (Days)	11.59 (6.18)	11.24 (4.99)	0.441	11.27 (5.04)	11.22 (6.65)	0.900
FIM Gain	28.76 (13.28)	32.84 (13.83)	0.001	30.67 (12.94)	31.98 (13.43)	0.172
FIM Gain per day	2.91 (1.91)	3.27 (1.89)	0.037	3.15 (1.79)	3.32 (1.85)	0.189
Return to Community (=1)	222 (87.4%)	225 (91.5%)	0.149	286 (84.1%)	380 (87.8%)	0.149
FIM Admission	62.21 (15.57)	64.12 (13.57)	0.159	67.90 (14.27)	66.98 (13.66)	0.342
Primary Payer (MCO = 1)	76 (29.9%)	119 (48.4%)	< 0.001	165 (48.5%)	272 (62.8%)	< 0.001
Case-Mixed Groups - 701 N (%)	21 (8.3%)	20 (8.1%)	0.219	39 (11.5%)	43 (9.9%)	0.918
CMG - 702	44 (17.3%)	56 (22.8%)		89 (26.2%)	120 (27.7%)	
CMG - 703	54 (21.3%)	63 (25.6%)		76 (22.4%)	100 (23.1%)	
CMG - 704	128 (50.4%)	99 (40.2%)		130 (38.2%)	160 (37.0%)	
CMG - 5001	7 (2.8%)	7 (2.8%)		6 (1.8%)	10 (2.3%)	
CMG - 5101	/	1 (0.4%)		/	0 (0.0%)	
Comorbidity Tier						
- None	154 (60.6%)	146 (59.3%)	0.081	232 (68.2%)	275 (63.5%)	0.421
- Minor	78 (30.7%)	91 (37.0%)		82 (24.1%)	115 (26.6%)	
- Moderate	14 (5.5%)	5 (2.0%)		13 (3.8%)	26 (6.0%)	
- Major	8 (3.1%)	4 (1.6%)		13 (3.8%)	17 (3.9%)	
Age (Years)	52.52 (11.53)	51.19 (12.36)	0.218	51.84 (11.79)	48.29 (13.56)	< 0.001
Gender (Female = 1)	156 (61.4%)	155 (63.5%)	0.627	197 (57.9%)	205 (47.5%)	0.004
Hispanic (=1)	16 (6.3%)	12 (4.9%)	0.504	31 (9.1%)	40 (9.2%)	0.946
Black (=1)	64 (25.2%)	50 (20.3%)	0.212	65 (19.1%)	81 (18.7%)	0.897
Marital Status						
- Married	56 (22.0%)	49 (19.9%)	0.832	65 (19.1%)	84 (19.4%)	0.015
- Never Married	109 (42.9%)	108 (43.9%)		166 (48.8%)	247 (57.0%)	
- Separated/Widowed	77 (30.3%)	80 (32.5%)		91 (26.8%)	92 (21.2%)	
- Missing	12 (4.7%)	9 (3.7%)		18 (5.3%)	10 (2.3%)	
No. of Certified Beds	52.61 (32.16)	56.39 (39.46)	0.257	47.84 (37.19)	52.75 (35.19)	0.057
Facility type						
- Freestanding	95 (37.4%)	/	< 0.001	103 (30.3%)	/	< 0.001
- In-Unit	159 (62.6%)	/		237 (69.7%)	/	
- Missing	/	246 (100.0%)		/	433 (100.0%)	
Joint Replacement						
N	418	275	~	709	626	~
Length of Stay (Days)	9.03 (3.88)	9.05 (3.62)	0.95	9.52 (4.61)	9.00 (3.90)	0.025
FIM Gain	31.31 (12.39)	34.13 (13.45)	0.005	31.80 (11.74)	30.52 (12.16)	0.056
FIM Gain per day	3.85 (1.86)	4.14 (2.00)	0.048	3.77 (1.66)	3.66 (1.63)	0.252
Return to Community (=1)	402 (96.2%)	258 (93.8%)	0.155	663 (93.4%)	578 (92.3%)	0.461
FIM Admission	71.60 (13.06)	70.46 (12.91)	0.262	72.93 (12.90)	75.33 (11.45)	< 0.001
Primary Payer (MCO = 1)	159 (38.0%)	154 (56.0%)	< 0.001	456 (64.2%)	491 (78.4%)	< 0.001
Case-Mixed Groups - 801 N (%)	10 (2.4%)	9 (3.3%)	0.925	28 (3.9%)	39 (6.2%)	0.048
CMG - 802	139 (33.3%)	87 (31.6%)		233 (32.8%)	238 (38.0%)	
CMG - 804	134 (32.1%)	87 (31.6%)		252 (35.5%)	214 (34.2%)	
CMG - 805	74 (17.7%)	53 (19.3%)		118 (16.6%)	78 (12.5%)	
CMG - 806	48 (11.5%)	33 (12.0%)		63 (8.9%)	43 (6.9%)	
CMG - 5001	13 (3.1%)	6 (2.2%)		16 (2.3%)	13 (2.1%)	
CMG - 5101	/	0 (0.0%)		/	1 (0.2%)	
Comorbidity Tier						
- None	268 (64.1)	144 (52.4%)	0.011	476 (67.0%)	368 (58.8%)	0.004
- Minor	145 (34.7%)	125 (45.5%)		224 (31.5%)	247 (39.5%)	
- Moderate	4 (1.0%)	6 (2.2%)		7 (1.0%)	11 (1.8%)	
- Major	1 (0.2%)	/		3 (0.4%)	/	
Age (Years)	53.75 (8.48)	54.63 (8.33)	0.183	53.88 (7.72)	54.67 (7.45)	0.062
Gender (Female = 1)	293 (70.1%)	193 (70.2%)	0.981	496 (70.0%)	384 (61.3%)	< 0.001
Hispanic (=1)	85 (20.3%)	15 (5.5%)	< 0.001	73 (10.3%)	77 (12.3%)	0.247
Black (=1)	149 (35.6%)	88 (32.0%)	0.322	224 (31.5%)	197 (31.5%)	0.961
Marital Status						
- Married	111 (26.6%)	50 (18.2%)	0.023	177 (24.9%)	162 (25.9%)	0.924
- Never Married	142 (34.0%)	120 (43.6%)		319 (44.9%)	280 (44.7%)	
- Separated/Widowed	156 (37.3%)	101 (36.7%)		191 (26.9%)	161 (25.7%)	
- Missing	9 (2.2%)	4 (1.5%)		23 (3.2%)	23 (3.7%)	
No. of Certified Beds	60.72 (33.02)	64.55 (42.83)	0.21	45.98 (38.68)	51.82 (35.57)	0.004
Facility type						
- Freestanding	128 (30.6%)	/	< 0.001	180 (25.4%)	/	< 0.001
- In-Unit	290 (69.4%)	/		530 (74.6%)	/	
- Missing	/	275 (100.0%)		/	626 (100.0%)	

Note: Entries for the categorical variables are number of observations with percentages in parentheses; entries for the continuous variables are the averages with standard errors in parentheses. * F-test was used to test the sample difference. *P*-value reported

The patterns of change in patient FIM score at admission and comorbidity mix across years were comparable between expansion and non-expansion states. However, proportionally fewer Black or Hispanic patients were admitted to IRF in the non-expansion states in 2016 than in 2013 (by about 5%). In contrast, proportionally equal shares of minority patients were admitted to IRF in the expansion states before and after the expansion. In addition, higher numbers of younger, male and never married patients were admitted to IRF in the expansion states in 2016 than 2013, which was in line with the expansion provisions, while patients in the non-expansion states showed no change in these demographics.

### Length of stay – resource utilization

Table 2 Column 2 shows the LOS results for all three impairment groups. Year and state differences were comparable except that stroke patients had shorter LOS by an average of 1.08 days (*P* < 0.05) in 2016 compared with 2013, and joint replacement patients in expansion states had longer LOS by 0.69 days (*P* < 0.01) than in non-expansion states. After controlling for year and state differences, the difference-in-difference estimates on the interaction terms suggested that patients experienced no significant change in LOS after expansion for any of the three impairment groups.

**Table 2:** Diff-in-Diff estimation of Medicaid effects on major outcome variables controlling all covariates

	FIM Admission	LOS	FIM GAIN	FIM Gain/day	ReturnToCommunity
**Stroke**					
Post (Year = 2016)	0.21 (0.39)	−1.08 (0.42)**	4.45 (0.61)***	0.41 (0.07)***	0.04 (0.02)***
Expansion (=1)	0.31 (0.31)	−0.45 (0.33)	−0.03 (0.48)	0.03 (0.05)	−0.08 (0.01)***
Post * Expansion	−0.31 (0.42)	0.67 (0.45)	−1.63 (0.66)**	− 0.21 (0.07)***	− 0.02 (0.02)
Medicare Advantage (=1)	0.01 (0.18)	−1.69 (0.19)***	− 0.98 (0.28)***	− 0.04 (0.03)	− 0.01 (0.01)
No. Obs.	11,619	11,619	11,619	11,619	11,619
**Fracture**					
Post (Year = 2016)	−1.30 (0.87)	−0.73 (0.52)	5.98 (1.18)***	0.53 (0.16)***	0.03 (0.03)
Expansion (=1)	2.29 (0.75)***	0.21 (0.45)	3.15 (1.02)***	0.27 (0.13)**	−0.06 (0.03)**
Post * Expansion	−0.97 (1.03)	0.18 (0.61)	−3.61 (1.40)**	−0.23 (0.18)	0.01 (0.04)
Medicare Advantage (=1)	1.11 (0.52)**	− 0.27 (0.31)	−0.64 (0.71)	− 0.04 (0.09)	− 0.03 (0.02)
No. Obs.	1270	1270	1270	1270	1270
**Joint Replacement**					
Post (Year = 2016)	−1.37 (0.58)**	− 0.19 (0.30)	4.28 (0.81)***	0.49 (0.12)***	−0.01 (0.02)
Expansion (=1)	0.05 (0.45)	0.69 (0.23)***	0.99 (0.63)	0.10 (0.09)	−0.04 (0.02)**
Post * Expansion	1.34 (0.68)**	−0.03 (0.35)	−2.73 (0.94)***	−0.44 (0.14)***	− 0.001 (0.02)
Medicare Advantage (=1)	0.88 (0.34)**	−0.57 (0.17)***	0.48 (0.47)	0.06 (0.07)	0.01 (0.01)
No. Obs.	2028	2028	2028	2028	2028
Control for …					
FIM Admission	~	Y	Y	Y	Y
LOS	~	~	Y	Y	Y
CMG	Y	Y	Y	Y	Y
Tier	Y	Y	Y	Y	Y
Demographics	Y	Y	Y	Y	Y
Facility Type	Y	Y	Y	Y	Y

* 90%, ** 95%, *** 99% significant level

- Entries are estimated beta coefficients with standard error in parentheses

### Functional outcomes - quality of care / care outcomes

Table 2 Column 3 shows that patients had significantly lower functional improvements in expansion states than non-expansion states after Medicaid expansion (i.e., the DinD term, by − 1.63, − 3.61, − 2.73 units, respectively; *P* < 0.05, 0.05 and 0.01). Average daily functional improvement (Table 2 Column 4) for stroke and joint replacement was also significantly lower in the expansion states after the reform (by − 0.21 and − 0.44 units/day; *P* < 0.01 for both). These results were obtained after controlling for the general year trend and the average difference between the expansion and non-expansion states.

### Return to community - health outcomes

After controlling for state and year differences, patients had no significant change in the likelihood of returning to community after discharge (Table 2 Column 5) after Medicaid expansion.

### Changes after Medicaid expansion by comorbidity group

Tables 3 and 4 show the results for the subgroups of patients without and with comorbidities.

**Table 3:** Estimation of Medicaid effects on major outcome variables for patient with no comorbidity

	FIM Admission	LOS	FIM GAIN	FIM Gain/day	ReturnCommunity
**Stroke**					
Post (Year = 2016)	−0.08 (0.51)	−1.56 (0.53)***	4.47 (0.77)***	0.49 (0.09)***	0.05 (0.02)**
Expansion (=1)	0.31 (0.38)	−0.46 (0.40)	0.05 (0.58)	0.02 (0.07)	−0.06 (0.02)***
Post * Expansion	−0.13 (0.54)	1.10 (0.56)*	−1.73 (0.82)**	−0.32 (0.10)***	− 0.04 (0.02)*
Medicare Advantage (=1)	0.01 (0.23)	−1.55 (0.24)***	−0.97 (0.35)***	− 0.04 (0.04)	− 0.01 (0.01)
No. Obs.	7148	7148	7148	7148	7148
**Fracture**					
Post (Year = 2016)	−2.28 (1.10)**	0.02 (0.52)	6.73 (1.39)***	0.62 (0.19)***	0.04 (0.04)
Expansion (=1)	1.69 (0.92)*	1.35 (0.44)***	4.44 (1.17)***	0.42 (0.16)***	−0.04 (0.03)
Post * Expansion	−0.07 (1.29)	−1.22 (0.61)**	−3.69 (1.62)**	− 0.25 (0.22)	0.0001 (0.04)
Medicare Advantage (=1)	1.12 (0.65)*	−0.25 (0.31)	−0.18 (0.82)	− 0.06 (0.11)	−0.01(0.02)
No. Obs.	805	805	805	805	805
**Joint Replacement**					
Post (Year = 2016)	−2.33 (0.77)***	−0.64 (0.37)*	2.26 (1.03)**	0.28 (0.16)*	−0.005 (0.02)
Expansion (=1)	−0.15 (0.58)	0.59 (0.28)**	0.17 (0.77)	0.01 (0.12)	−0.03 (0.02)
Post * Expansion	2.20 (0.90)**	0.36 (0.43)	−1.60 (1.19)	−0.32 (0.19)*	− 0.01 (0.03)
Medicare Advantage (=1)	0.73 (0.44)	− 0.48 (0.21)**	0.96 (0.59)	0.13 (0.09)	0.01 (0.01)
No. Obs.	1255	1255	1255	1255	1255
Control for …					
FIM Admission	~	Y	Y	Y	Y
LOS	~	~	Y	Y	Y
CMG	Y	Y	Y	Y	Y
Tier	Y	Y	Y	Y	Y
Demographics	Y	Y	Y	Y	Y
Facility Type	Y	Y	Y	Y	Y

* 90%, ** 95%, *** 99% Significant Level

- Entries are estimated beta coefficients with standard error in parentheses

**Table 4:** Estimation of Medicaid effects on major outcome variables for patient with comorbidities

	FIM Admission	LOS	FIM GAIN	FIM Gain/day	ReturnCommunity
**Stroke**					
Post (Year = 2016)	0.65 (0.61)	−0.28 (0.71)	4.45 (1.03)***	0.33 (0.10)***	0.04 (0.03)
Expansion (=1)	0.31 (0.52)	−0.41 (0.60)	−0.11 (0.87)	0.04 (0.09)	−0.10 (0.02)***
Post * Expansion	−0.52 (0.66)	0.15 (0.77)	−1.48 (1.11)	−0.09 (0.11)	0.01 (0.03)
Medicare Advantage (=1)	−0.02 (0.28)	−2.03 (0.33)***	−0.98 (0.48)**	− 0.04 (0.05)	−0.01 (0.01)
No. Obs.	4471	4471	4471	4471	4471
**Fracture**					
Post (Year = 2016)	0.23 (1.44)	−1.66 (1.07)	4.70 (2.16)**	0.46 (0.26)*	0.02 (0.06)
Expansion (=1)	3.23 (1.30)**	−1.77 (0.97)*	1.08 (1.97)	0.28 (0.24)	−0.11 (0.05)**
Post * Expansion	−2.37 (1.75)	2.43 (1.30)*	−3.55 (2.64)	−0.51 (0.32)	0.03 (0.07)
Medicare Advantage (=1)	1.24 (0.90)	−0.14 (0.67)	−0.94 (1.36)	0.02 (0.17)	−0.06 (0.04)
No. Obs.	465	465	465	465	465
**Joint Replacement**					
Post (Year = 2016)	0.48 (0.91)	0.45 (0.52)	6.89 (1.34)***	0.72 (0.18)***	−0.01 (0.03)
Expansion (=1)	0.77 (0.74)	0.82 (0.43)*	1.82 (1.09)*	0.17 (0.14)	−0.05 (0.03)*
Post * Expansion	−0.17 (1.05)	−0.43 (0.60)	−3.49 (1.54)**	− 0.47 (0.20)**	0.02 (0.04)
Medicare Advantage (=1)	1.15 (0.54)**	−0.84 (0.31)***	−0.46 (0.80)	− 0.07 (0.10)	0.003 (0.02)
No. Obs.	773	773	773	773	773
Control for …					
FIM Admission	~	Y	Y	Y	Y
LOS	~	~	Y	Y	Y
CMG	Y	Y	Y	Y	Y
Tier	Y	Y	Y	Y	Y
Demographics	Y	Y	Y	Y	Y
Facility Type	Y	Y	Y	Y	Y

* 90%, ** 95%, *** 99% Significant Level

- Entries are estimated beta coefficients with standard error in parentheses

For stroke, among those patients with no comorbidities (Table 3), LOS was marginally longer (by 1.10 days, *P* < 0.10), FIM gain was 1.73 units lower (*P* < 0.05), daily FIM gain was 0.32 units/day lower (*P* < 0.01) and possibility of returning to community was 4% lower (*P* < 0.10) in expansion states than in non-expansion states after Medicaid expansion. In comparison, patients with comorbidities (Table 4) did not experience significant differences between expansion and non-expansion states after Medicaid expansion.

For hip fracture, patients with no comorbidities experienced significantly lower LOS (− 1.22 days, *P* < 0.05) and lower FIM score improvement (− 3.69 units, *P* < 0.05) in expansion states than in non-expansion states after Medicaid expansion, while patients with pre-existing comorbidities experienced a marginally significant increase in LOS (2.43 days, *P* < 0.10) and no significant changes for functional improvement.

For joint replacement, an elective condition, patients with comorbidities had significantly lower functional improvement (− 3.49 units, *P* < 0.05) in the expansion states than in non-expansion states after the expansion. However, patients without comorbidities did not experience significant differences.

Comparing across the three impairment groups, results suggested that patients with pre-existing conditions admitted to inpatient rehabilitation services due to non-deferrable reasons (e.g., stroke and fracture) benefitted more from Medicaid expansion than those without any health conditions. However, for a relatively deferrable treatment such as joint replacement, those patients without pre-existing conditions who “self-selected” to the treatment after the expansion benefitted more than those with pre-existing conditions.

## Discussion

### Main findings and contribution

Utilization of rehabilitation services in IRF increased after Medicaid expansion, particularly among minority subpopulations (Hispanic or Black). Patient composition of diagnosis groups and comorbidity tiers were not significantly changed. Patients with no comorbidities in the acute conditions (i.e., stroke and hip fracture) and patients with comorbidities in the elective condition (i.e., joint replacement) had lower functional improvements in the expansion states than in the non-expansion states after the reform. Average length of stay and possibility of returning to community were not changed significantly.

This study is unique to the literature and contributes to: (1) the rehabilitation services research among low-income and socio-economically disadvantaged Medicaid beneficiaries as opposed to Medicare and other commercial insurance recipients [18, 31–33] and on non-elderly adults, a population absent in previous studies [23, 26, 34, 35]; (2) the health policy evaluations on patient functional improvements as the direct health outcomes [34]; (3) the concurrent debates regarding the mixed findings of Medicaid expansion effects [9] such as reduction in treatment costs (and quality) or shortened inpatient length of stay due to service pressure after volume expansion [32, 33]; and (4) the PAC service in IRF under the US ACA among non-elderly adult patients with the three most common conditions for inpatient rehabilitation other than trauma (e.g. stroke, hip fracture and joint replacement, etc.), which has not been studied thus far [16, 17].

In particular, the study contributes to the discussion of the mixed findings on the effects of Medicaid expansion on healthcare utilization and quality outcomes in general by interpreting the results through alternative angles and comparison groups [9, 12, 32]. For example, due to the policy background of increasing reimbursement restrictions and cost control efforts, service volume of post-acute rehabilitation has decreased substantially over the past decade [15]. As a result, the total episodes for some treatments, such as joint replacement, decreased after the expansion. However, comparison with the time trend for non-expansion states revealed that Medicaid expansion states experienced much smaller reduction in service volume over the study period, suggesting positive effects of the expansion on care utilization.

Additionally, the average FIM score at admission for certain patient groups reflected the admission restrictions or priorities for IRF due to the stringent reimbursement policies [36, 37]. However, the underlying interpretation of these scores depended on the specific treatment under discussion. For the essential and non-deferrable conditions such as stroke and hip fracture, priorities tended to be given to those patients with better tolerance (i.e., higher functional status at admission) in order to receive the rehabilitation therapy more successfully. Yet, for elective treatments such as joint replacement, priorities were given to those patients with more urgent needs (i.e., lower functional status at admission). With this clinical background in mind, both decrease in FIM score at admission for stroke and hip fracture and increase in FIM score at admission for joint replacement implied positive effects of Medicaid expansion on care utilization, as shown on the interaction terms in Table 2 Column 2 [36, 37].

Moreover, results showed that functional improvements for stroke and joint replacement were lowered after expansion. Stratifying the study sample by patients with and without comorbidities provided further insights on this seemingly negative evidence. It was shown that lower functional improvements were only limited to patients with no comorbidities for stroke and hip fracture, and patients with comorbidities for joint replacement. On possible explanation is that those who were more functional limited became Medicaid eligible in the expansion states. For the non-deferrable acute conditions such as stroke or fracture, these newly eligible beneficiaries were more likely to be patients with no comorbidity. For elective conditions, when more patients self-selected into a procedure (e.g., joint replacement) due to expansion in coverage, those with pre-existing health conditions were at higher risk of being worse off than those without pre-existing conditions once IRF were facing resource constraints due to the sudden increases in service volume [32].

Lastly, though the estimated differences in LOS and functional improvements associated with the ACA Medicaid expansion were small in the absolute values, they represented almost 6% differences relative to the average value (e.g., 1.76 points lower in functional improvement relative to an average of 28.77 points in total improvement for stroke), which is considered practically significant. Further, these results merit for practical attention for two additional reasons. On the one hand, even smaller percentage differences in care delivery can yield larger differences in the subsequent and long-term outcomes due to the potential leverage power. On the other hand, due to the large population base of Medicaid and the high costs of daily inpatient stay in the US, even 1% difference per patient in the current or future resource utilization can yield huge cost differences in total.

### Limitations and future research

The study also had limitations.

First, including states with early Medicaid expansion, such as Massachusetts and New York, in the expansion states could underestimate the expansion effects (i.e., lower the point estimates). Though sensitivity analysis in the study by including or excluding early expansion states and by weighting the results by Medicaid enrollment numbers and/or percentage changes showed no significant differences in the main results, categorizing the patient records by specific treatment year-month and state-level expansion year-month could yield more accurate estimates with higher generosity.

Second, there were concerns about sample representativeness. According to CMS, patient documentation using the IRF-PAI and FIM instrument was only required for traditional Medicare Fee-for-Service (FFS) beneficiaries during the reimbursement procedure but not for Medicaid beneficiaries [20]. If at least some IRF chose not to document patient records for non-Medicare FFS patients, then the study sample would not be representative. This concern can be alleviated if all CMS qualified IRF documented their patient records in the same way regardless of patient insurance types, which, for administration purposes, is likely the case for most IRF [27]. On the other hand, due to its means-test property (i.e. eligibility ties to income and wealth level), Medicaid has large turnover rates from year to year, and, therefore, the beneficiaries may not be fully comparable over time [7, 38]. As many studies on the same topic have noted, the eligibility of Medicaid expansion is random and exogenous, but the enrollment and actual utilization are not [7]. This is the case with most studies on Medicaid expansion, except for very few, such as the Oregon study series [38].

Third, the study had a short period of observation and only used the year 2013 as the pre-expansion comparison and 2016 as the post-expansion outcomes year. As Medicaid expansion has been adopted by more and more states over time and multiple years of evidence becomes available, it is beneficial to conduct a state, year-level study to show the dynamics and the timing of expansion effects. Longer time horizon will also help to discern the short-term negative effects of expansion due to the sudden increase in service volume.

Finally, rather than claiming the estimates as unbiased policy effects in isolation of any time variant unobservable as other typical difference-in-difference studies would do, estimates in this study implied the net effects of expansion within the background of multiple policy/institutional changes over the study period (e.g., service volume reduction across the board, yet lower reduction among the expansion regions). The inherent endogeneity problems within the expansion states (the treatment group) still remained. Some of the observed changes might result from the changes in patient/beneficiary composition beyond the control variable that we could adjust in the study. For example, more functionally limited patients would become newly eligible under the Medicaid expansion for acute care at first and inpatient rehabilitation thereafter. They may not be able to come up to the level of function that was seen in the non-expansion beneficiaries. Understanding the changes in the beneficiary pool, especially in terms of their functionality even before the acute care treatment (not at the time of inpatient rehabilitation admission) would provide more insights. Similarly, additional studies on the changes among the non-insured population over the same period would also help.

## Conclusions

In summary, this study showed that Medicaid expansion increased post-acute care utilization in IRF, especially among minority groups. Cautions should be taken when one tried to interpret some seemingly insignificant or even negative care outcomes, such as lower overall functional improvements and daily average improvements, which were associated with treatment types and pre-existing conditions and possibly due to the rehabilitation service volume constraints right after the expansion. A longer time frame and more refined scale for modeling may provide more valuable insights on the relative cost and quality of the services.

## Figures and Tables

**Table 5:**
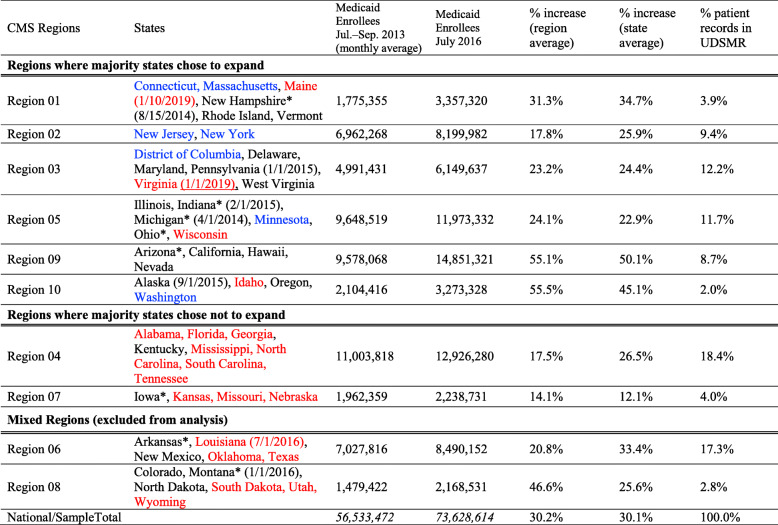
CMS regions with state expansion status, Medicaid beneficiary counts, % increases and UDSMR sample distributions

The effective date of expansion is January 1, 2014 unless otherwise noted in parenthesis

States WITHOUT Medicaid expansion as of January 1, 2016 are marked in **Red**

States with EARLY Medicaid expansion before January 1, 2014 are marked in **Blue**

Note: Expansion regions showed higher percentage increases in Medicaid enrollments than non-expansion regions. Expansion region 01, 02 and 03 had lower percentage increases than other expansion regions since some states had early expansions before 2014 (e.g., New York) and/or some populous states with late expansion after 2016 (e.g., Virginia)

* means the state is approved with Section 1115 waivers

## Data Availability

The data that support the findings of this study is available from the Uniform Data System for Medical Rehabilitation (UDSMR), but restrictions apply to the availability of these data, which were used under license for the current study, and so are not publicly available. Data are however available from the authors upon reasonable request and with permission of UDSMR.

## Appendix B

**Table 6:** CMS Categorization of the Case Mix Groups (CMG) (M-Motor, C-Cognitive, A-Age)

Stroke		Hip Fracture		Joint Replacement	
CMG101	Stroke M > 51.05	CMG701	Fracture of lower extremity M > 42.15	CMG801	Replacement of lower extremity joint M > 49.55
102	Stroke M > 44.45 and M < 51.05 and C > 18.5	702	Fracture of lower extremity M > 34.15 and M < 42.15	802	Replacement of lower extremity joint M > 37.05 and M < 49.55
103	Stroke M > 44.45 and M < 51.05 and C < 18.5	703	Fracture of lower extremity M > 28.15 and M < 34.15	803	Replacement of lower extremity joint M > 28.65 and M < 37.05 and A > 83.5
104	Stroke M > 38.85 and M < 44.45	704	Fracture of lower extremity M < 28.15	804	Replacement of lower extremity joint M > 28.65 and M < 37.05 and A < 83.5
105	Stroke M > 34.25 and M < 38.85	5001	Short-stay cases, length of stay is 3 days or fewer	805	Replacement of lower extremity joint M > 22.05 and M < 28.65
106	Stroke M > 30.05 and M < 34.25	5101	Expired, orthopedic, length of stay is 13 days or fewer	806	Replacement of lower extremity joint M < 22.05
107	Stroke M > 26.15 and M < 30.05			5001	Short-stay cases, length of stay is 3 days or fewer
108	Stroke M < 26.15 and A > 84.5			5101	Expired, orthopedic, length of stay is 13 days or fewer
109	Stroke M > 22.35 and M < 26.15 and A < 84.5				
110	Stroke M < 22.35 and A < 84.5				
5001	Short-stay cases, length of stay is 3 days or fewer				

## Abbreviations

ACA: Patient Protection and Affordable Care Act (or PPACA)
CMG: Case-mix Group
CMS: Center for Medicare & Medicaid Services
FIM: Functional Independence Measure
ICD-9: International Classification of Diseases, Ninth Revision
IRF: Inpatient Rehabilitation Facilities
IRF-PAI: Inpatient Rehabilitation Facilities – Patient Assessment Instrument
LOS: Length of Stay
PAC: Post-acute Care
UDSMR: Uniform Data System for Medical Rehabilitation
